# Pulmonary Metastasectomy for Colorectal Cancer: Evidence and Outcomes—A Narrative Review

**DOI:** 10.3390/jcm14124172

**Published:** 2025-06-12

**Authors:** Athanasios Papatriantafyllou, Konstantinos Grapatsas, Francesk Mulita, Nikolaos G. Baikoussis, Elias Liolis, Levan Tchabashvili, Konstantinos Tasios, Spyros Papadoulas, Manfred Dahm, Vasileios Leivaditis

**Affiliations:** 1 Department of Cardiothoracic and Vascular Surgery, Westpfalz Klinikum, 67655 Kaiserslautern, Germany; thanospap9@yahoo.gr (A.P.); mdahm@westpfalz-klinikum.de (M.D.); vnleivaditis@gmail.com (V.L.); 2 Department of Thoracic Surgery and Thoracic Endoscopy, Ruhrlandklinik, West German Lung Center, University Hospital Essen, University Duisburg-Essen, 45141 Essen, Germany; grapatsaskostas@gmail.com; 3 Department of General Surgery, General Hospital of Eastern Achaia—Unit of Aigio, 25100 Aigio, Greece; med5507@upnet.gr (F.M.); tchabashvili.alexander@gmail.com (L.T.); 4 Department of Cardiac Surgery, Ippoktration Gernaral Hospital of Athens, 11527 Athens, Greece; nikolaos.baikoussis@gmail.com; 5 Department of Oncology, University Hospital of Patras, 26504 Patras, Greece; lioliselias@yahoo.gr; 6 Department of General Surgery, General University Hospital of Patras, 26504 Patras, Greece; kostastasiosmd@gmail.com; 7 Department of Vascular Surgery, University Hospital of Patras, 26504 Patras, Greece

**Keywords:** colorectal cancer, pulmonary metastases, pulmonary metastasectomy, prognostic factors, surgical outcomes, survival

## Abstract

Pulmonary metastasectomy for colorectal cancer represents a key component in modern oncological surgery, balancing precision resection with systemic disease management. Despite ongoing debate initiated by randomized trials, the surgical removal of lung metastases continues to offer significant survival benefits in well-selected patients. This review synthesizes the evolving landscape of pulmonary metastasectomy, integrating classical prognostic indicators, such as the disease-free interval (DFI) and carcinoembryonic antigen (CEA) levels, with emerging molecular insights including KRAS and BRAF mutations. The relationship between surgical radicality, systemic therapies, and personalized genetic profiling is redefining patient selection and optimizing outcomes. By dissecting recent evidence and ongoing controversies, we clarify the complex decision-making required to navigate this complex clinical terrain. Ultimately, the synergy of multidisciplinary care and precision surgery holds the promise of durable disease control and extended survival in colorectal cancer patients with lung metastases.

## 1. Introduction

Colorectal cancer (CRC) is the third most common malignancy worldwide and is a leading cause of cancer-related mortality [1]. Approximately 10–15% of patients with CRC will develop pulmonary metastases, often as part of systemic disease progression [2,3]. Patients with CRC frequently face the development of lung metastases, which represent a systemic manifestation of the disease and signify an advanced stage with complex therapeutic challenges [2,3,4]. While systemic therapies such as chemotherapy and targeted treatments remain essential, pulmonary metastasectomy has emerged as a promising therapeutic option, particularly after successful resection of the primary colorectal tumor [1,4]. Whether performed synchronously with the primary tumor resection or as a subsequent metachronous procedure, the surgical removal of lung metastases offers a potential path to long-term survival, and in select cases, a cure [1,5]. The initial step in managing pulmonary metastases involves a rigorous assessment of resectability, taking into account a range of clinical and technical factors alongside the patient’s overall health status [6]. This review aimed to analyze the most significant factors affecting the decision-making process for pulmonary metastasectomy in colorectal cancer patients and evaluate the impact of this intervention on patient survival.

## 2. Materials and Methods

A comprehensive literature review was conducted to evaluate the outcomes and prognostic factors associated with pulmonary metastasectomy in colorectal cancer patients. Searches were performed across major databases, including PubMed, Scopus, Cochrane Library, and Embase, to identify relevant peer-reviewed studies published in English. The search encompassed the literature from January 1995 to March 2025, although the vast majority of included studies were published after 2010. The search strategy incorporated a combination of MeSH terms and free-text keywords, such as “colorectal cancer”, “colorectal neoplasms”, “lung metastases”, “pulmonary metastases”, “pulmonary metastasectomy”, “prognostic factors”, and “surgical outcomes”, which were combined using Boolean operators (AND, OR) to optimize sensitivity and specificity, and the following core terms and their combinations were applied: (“colorectal cancer” OR “colorectal neoplasms”) AND (“lung metastases” OR “pulmonary metastases”) AND “pulmonary metastasectomy” AND “prognostic factors” AND “surgical outcomes”. Studies were included if they reported on patient outcomes, prognostic indicators, or surgical techniques related to lung metastasectomy. Exclusion criteria included studies involving non-human subjects, non-English publications, and those lacking sufficient data on the survival or treatment outcomes.

**Figure 1:**
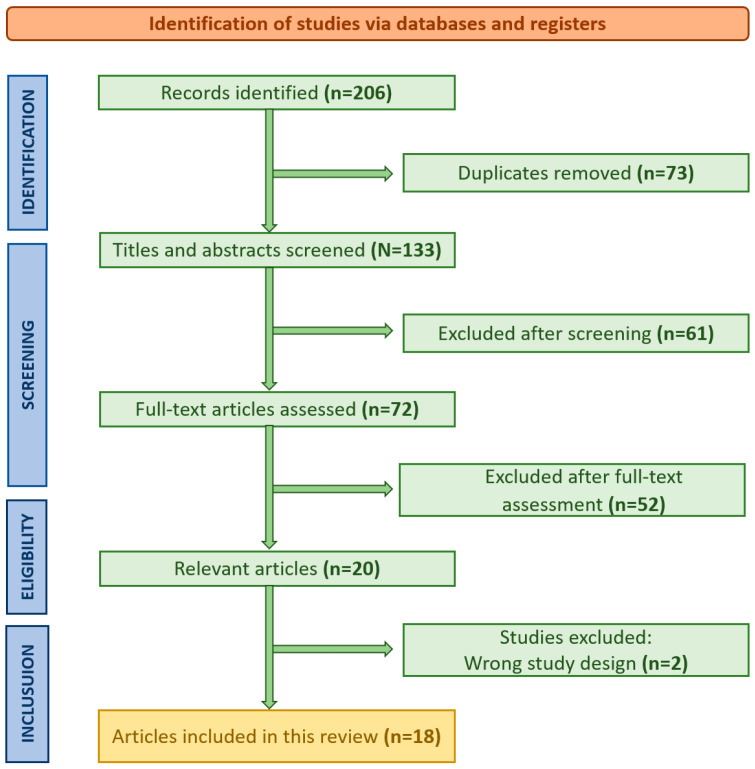
PRISMA (Preferred Reporting Items for Systematic Reviews and Meta-Analyses) flowchart illustrating the study selection process for this review.

The initial search retrieved a total of 206 studies across all databases. Of these, 18 studies met all of the inclusion criteria and were included in the final review. The most common reasons for exclusion were: non-English language, lack of outcome data (e.g., survival, recurrence), non-human subjects, and irrelevant focus (e.g., primary CRC management without reference to pulmonary metastases). A summary of the study selection process is provided in the flow diagram in Figure 1. While 18 studies were included in the comparative synthesis table, additional references were cited throughout the manuscripts to support specific subtopics.

One of the main limitations encountered during the literature search was the heterogeneity of the study designs and outcome measures. Several studies lacked standardized definitions for key prognostic factors such as disease-free interval or resection margins, limiting direct comparability. Additionally, publication bias and the predominance of retrospective data may have influenced the strength of some conclusions.

The final set of included studies was predominantly published after 2010, with publication years ranging from 1995 to 2025. Studies originated from a broad range of countries including European, American, and Asian countries. This geographic diversity strengthens the generalizability of the review findings.

## 3. Interdisciplinary Approach and Treatment Efficacy

Achieving the optimal outcomes for colorectal cancer patients with pulmonary metastases requires a multidisciplinary approach involving collaboration among surgeons, oncologists, radiologists, and other specialists. Tumor board discussions play a crucial role in formulating individualized treatment plans, which consider critical factors such as the timing of metastasis detection (synchronous vs. metachronous), the patient’s prior systemic therapy history, and personal treatment preferences [1,6]. These multidisciplinary consultations are essential to evaluate the suitability of surgical intervention and adjust strategies for both metastatic and palliative cases [2,4].

The efficacy of treatment modalities, including surgery, chemotherapy, and targeted therapies, largely depends on patient-specific clinical indications. Surgical resection, particularly when combined with (neo)adjuvant chemotherapy or minimally invasive techniques (like VATS), has demonstrated significant promise in enhancing oncological outcomes [7,8,9].

Advances in tailored surgical approaches, including subsegmental and anatomical resections, have expanded the range of candidates for metastasectomy, enabling treatment for patients with initially inoperable lung metastases and offering potential curative outcomes [10,11].

## 4. Prognostic Factors

Key prognostic factors influencing outcomes in patients undergoing pulmonary metastasectomy for colorectal cancer (CRC) include the disease-free interval (DFI) and preoperative carcinoembryonic antigen (CEA) levels, both of which are widely recognized as independent indicators of tumor burden and key guides for treatment planning [7,12,13].

Additional critical parameters include the number, size, and anatomical distribution of lung metastases, which significantly impact survival [11,14,15]. Studies have demonstrated that solitary and unilateral metastases are associated with better prognosis, while multiple or bilateral lesions tend to lead to worse outcomes [16,17].

Furthermore, while patient demographics such as age and gender have been investigated as potential prognostic factors, most studies have not demonstrated a clear impact on the survival outcomes [14]. In contrast, the extent and radicality of metastatic resection remain determinants of prognosis, with radical (R0) resection associated with improved long-term survival [10,18,19]. Achieving negative surgical margins and complete tumor clearance continues to represent the key element of effective pulmonary metastasectomy.

### 4.1. Disease-Free Interval

The disease-free interval (DFI) is defined as the time between the surgical removal of the primary colorectal tumor and the initial clinical or radiological detection of recurrence. A prolonged DFI following primary colorectal cancer (CRC) surgery has traditionally been linked to improved survival outcomes, although its prognostic significance remains debated. The International Registry of Lung Metastases (IRLM), established by the European Society of Thoracic Surgeons (ESTS), was one of the first to emphasize that patients with a DFI of three years or more demonstrated a survival advantage [20]. This has been confirmed by subsequent studies, reporting tumor-free intervals ranging from 12 to 60 months as favorable [21,22,23,24]. A large-scale study by Cho et al. involving 615 CRC patients undergoing pulmonary metastasectomy found that the median DFI was 20 months [15], reinforcing the variability in DFI among patients. Prolonged DFIs often indicate lower tumor biological aggressiveness, reflected in longer tumor doubling times (TDTs). Conversely, shorter DFIs are associated with more aggressive tumor behavior and poorer survival outcomes.

However, despite the extensive body of evidence, the predictive value of DFI remains inconsistent, and it is considered unreliable as a single criterion for metastasectomy eligibility. Notably, the emergence of pulmonary metastases within 12 months of primary tumor resection is not a contraindication for surgery based on current evidence [6].

Importantly, a recent meta-analysis by Gkikas et al. found that neither DFI < or >24 months nor synchronous versus metachronous metastasis had a significant impact on postoperative survival [16]. This suggests that while DFI remains an important factor, it should be integrated within a broader clinical assessment.

### 4.2. CEA Levels

Carcinoembryonic antigen (CEA) is a key biomarker in colorectal cancer (CRC), widely used for monitoring disease progression and assessing tumor burden. In the context of pulmonary metastasectomy, preoperative CEA levels have been routinely evaluated as a prognostic indicator. Studies have shown that patients with normal preoperative CEA levels tend to achieve superior five-year survival rates, ranging from 23% to 80%, while those with elevated CEA still demonstrate promising survival outcomes, reaching up to 53% [12,25]. Importantly, elevated serum CEA should not preclude surgical intervention, as high levels may simply reflect a solitary large metastasis rather than extensive metastatic spread. However, it is important to note that not all colorectal tumors produce CEA. In such cases, CEA cannot be used as a biomarker for tumor burden, and its absence does not necessarily indicate limited disease [26,27]. Beyond its prognostic value, CEA remains a key tool in postoperative follow-up, enabling the early detection of recurrence and facilitating timely therapeutic interventions [2]. Its role in ongoing patient monitoring is especially critical, as changes in the CEA levels can indicate the need for further diagnostic imaging or therapeutic adjustments.

CEA is expressed in approximately 70% to 90% of CRC tumors, making it a valuable but imperfect marker not only for recurrence, but also for assessing treatment response [27]. Its expression continues to be studied in various clinical contexts, including novel applications such as intraoperative detection and guidance technologies [28]. While lower preoperative CEA is generally associated with better survival outcomes, elevated levels should not automatically exclude patients from metastasectomy, particularly when other factors (such as solitary lesions, favorable DFI, or good performance status) are present [2,29].

### 4.3. Age and Gender

The potential impact of age and gender on the survival outcomes following pulmonary metastasectomy (PM) for colorectal cancer (CRC) has been thoroughly investigated across large-scale studies conducted over the past two decades. Gender has not emerged as an independent prognostic factor, with the survival rates equivalent between men and women undergoing metastasectomy [4,14].

Similarly, patient age has not demonstrated a significant prognostic impact in most analyses. Extensive cohort studies have failed to establish a clear correlation between age and survival following PM [3,14]. However, one study by Iizasa et al. reported a potential survival advantage in patients aged over 60 years, though this finding has not been consistently replicated in subsequent research. The authors hypothesized that this paradoxical survival benefit might relate to selection bias, as older patients chosen for surgery may represent a particularly fit subgroup [14].

### 4.4. Performance Status and Comorbidities

Patient-related factors, particularly performance status and comorbidities, play a critical role in the prognostic assessment and surgical decision-making for pulmonary metastasectomy. Impaired performance status, commonly evaluated using the ECOG or Karnofsky scales, has been independently associated with poorer overall survival following metastasectomy [2,19,30]. Similarly, the presence of significant comorbidities—especially cardiovascular or pulmonary—can substantially increase the perioperative risk and may contraindicate surgical intervention altogether [4,19,31].

### 4.5. Primary Tumor Characteristics

The pathological features of the primary colorectal tumor—specifically T stage, N stage, and histologic grade—are strongly associated with metastatic potential and long-term survival following PM. High T stage and nodal involvement have been linked to shorter disease-free intervals and an increased likelihood of recurrence after lung resection. Additionally, poorly differentiated tumors tend to exhibit more aggressive biological behavior and worse prognosis [29,30,32].

### 4.6. Molecular and Genetic Markers

Beyond traditional clinical and pathological factors, molecular and genetic markers play a key role in prognostication and treatment planning for colorectal cancer (CRC) patients undergoing pulmonary metastasectomy. Among the most extensively studied are the KRAS, BRAF, and p53 mutations as well as microsatellite instability (MSI) status. KRAS mutations, present in approximately 40–50% of CRC cases, have been associated with worse overall survival (OS) and recurrence-free survival (RFS) following pulmonary metastasectomy. A meta-analysis of more than 15,000 patients demonstrated that KRAS mutations were significantly associated with poorer overall survival (OS) and disease-free survival (DFS), with the negative prognostic impact being particularly pronounced in microsatellite-stable (MSS) tumors [33]. BRAF mutations, though less frequent (5–10%), are linked to aggressive tumor behavior, right-sided primaries, and poorer outcomes. The RAXO study highlighted that BRAF-mutant patients tended to have lower resectability rates and worse survival post-metastasectomy, raising questions about the true benefit of surgery in this subgroup [34]. p53 mutations have also been recognized as potential markers of poor prognosis, with some studies suggesting that their presence correlates with shorter survival post-metastasectomy [16].

Additionally, microsatellite instability-high (MSI-H) status, while generally rare in metastatic CRC, may offer prognostic advantages due to its association with better immunogenicity and response to immune checkpoint inhibitors. However, its exact role in guiding surgical decisions for lung metastases is still being studied. Integrating molecular profiling into clinical decision-making will enable a more personalized approach, identifying high-risk patients who may benefit from alternative or additional therapies apart from metastasectomy alone [34]. As research continues to evolve, these markers may further refine the patient selection criteria and treatment algorithms.

### 4.7. Response to Systemic Therapy

The tumor’s response to neoadjuvant or perioperative chemotherapy serves as a valuable prognostic marker. Favorable radiologic or biochemical responses are associated with improved overall survival, reflecting the underlying tumor chemosensitivity. In contrast, progression during systemic therapy may indicate more aggressive disease biology, even if technically resectable [7,34].

### 4.8. Number, Size and Location of Metastases

The number, size, and spatial distribution of pulmonary metastases are decisive factors in determining prognosis and guiding surgical decision-making for patients with colorectal cancer (CRC) lung metastases. These technical aspects of resection have been thoroughly studied, though their prognostic implications vary across the literature. A widely accepted classification differentiates between solitary and multiple metastases. Patients with a solitary lung metastasis generally exhibit better survival outcomes [14,22]. However, the presence of multiple metastases does not preclude surgery, provided that a radical (R0) resection is technically feasible, thereby emphasizing the importance of complete resection over the number of lesions [3].

In terms of size, the maximum diameter of resected metastases is another critical but controversial factor. Various studies have applied different size cutoffs, typically ranging from 10 mm to 50 mm, complicating efforts to standardize its prognostic significance. Only a few studies, including Iizasa et al. and Vogelsang et al., have identified diameters exceeding 30–37.5 mm as independently associated with worse survival outcomes [14,35]. However, the prognostic impact of size appears to be inconsistent across studies, and most have not demonstrated a clear survival advantage for patients with smaller lesions. This variability suggests that the tumor size alone may not adequately reflect the tumor biology or aggressiveness. As such, size should be interpreted in the context of other clinical parameters including disease-free interval, number of metastases, and molecular characteristics [16].

The anatomical distribution of metastases—specifically unilateral versus bilateral lung involvement—also plays a significant role in prognosis. While bilateral metastases tend to correlate with poorer survival, unilateral disease has been associated with better outcomes in select studies [33,36]. This has been further supported by recent analyses that reinforce the prognostic impact of anatomical distribution in survival outcomes [37]. Nevertheless, the prognostic impact of laterality remains an area requiring further investigation to fully understand its role in clinical decision-making.

Survival outcomes correlate inversely with the number and size of the metastases. Patients with solitary or unilateral metastases generally experience superior survival, whereas those with bilateral disease or lesions larger than 2 cm face poorer prognoses [16,38]. However, radical resection remains feasible and potentially beneficial, even in the presence of multiple metastases, provided that complete resection can be achieved [3,22].

### 4.9. Laterality of Lung Metastases

Pulmonary disease laterality also carries prognostic significance. Bilateral pulmonary metastases are generally associated with a greater metastatic burden and have been linked to significantly worse overall survival when compared with unilateral disease [4,18,29]. However, in appropriately selected patients, bilateral pulmonary metastasectomy may still achieve satisfactory long-term outcomes, especially when favorable prognostic features such as a prolonged disease-free interval, limited number and size of nodules, and preserved pulmonary function are present [3,5,18,30].

### 4.10. Resection Extent and Lymphadenectomy

The extent of surgical resection is a determinant of outcomes in pulmonary metastasectomy (PM) for colorectal cancer (CRC). Surgical strategies typically range from subsegmental resections to more extensive anatomical resections including segmentectomy, lobectomy, or in rare cases, pneumonectomy. Subsegmental (wedge) resections are generally preferred due to their ability to preserve lung function in patients with limited pulmonary reserve or those who may require repeated metastasectomies [11]. However, anatomical resections may be necessary for centrally located or larger lesions, where negative surgical margins cannot be otherwise ensured [10].

Achieving negative resection margins (R0) is strongly associated with improved survival outcomes. Studies support that maintaining at least a 2 cm parenchymal margin, when feasible, significantly reduces the risk of local recurrence and improves long-term survival [10,11]. The choice of resection technique must be individualized, balancing the need for complete tumor clearance with the preservation of adequate lung function.

The role of lymphadenectomy during PM remains controversial. Some advocate for systematic lymph node dissection, citing its prognostic value in identifying nodal metastases [12]. Conversely, others favor a selective approach, limiting lymphadenectomy to cases where preoperative imaging or intraoperative findings suggest nodal involvement [4]. Recent studies have supported the prognostic significance of lymph node involvement, supporting the role of lymphadenectomy for staging purposes [39].

Regardless of the approach, there is strong evidence that hilar (N1) or mediastinal (N2) lymph node involvement is associated with significantly poorer survival outcomes in patients with mCRC [3,4]. Given these findings, accurate nodal assessment—whether through preoperative staging (e.g., PET-CT, EBUS) or intraoperative sampling—is essential for optimizing patient selection and prognosis.

In cases where the intraoperative frozen section reveals positive lymph nodes, the decision to proceed with pulmonary metastasectomy remains complex and must be individualized. While nodal positivity (N1 or N2) is associated with poor prognosis, some centers still advocate for resection in selected patients with favorable overall clinical profiles. Importantly, the presence of unexpected nodal involvement intraoperatively should prompt a reassessment of the surgical plan but not universally preclude resection. Further studies are needed to better define criteria for intraoperative decision-making in such scenarios [3,4,39].

### 4.11. Radicality

The radicality of resection, defined by the achievement of clear surgical margins (R0 resection), is widely recognized as the most critical prognostic factor in the surgical management of pulmonary metastases from colorectal cancer (CRC). Multiple studies have demonstrated that the complete resection of all detectable metastatic lesions significantly improves the survival outcomes [40,41,42]. In contrast, incomplete resections—classified as R1 (microscopically positive margins) or R2 (macroscopically incomplete resections)—are associated with substantially worse prognoses, with marked reductions in 5-year survival rates. To ensure optimal outcomes, a minimum margin of 5 mm of healthy lung parenchyma around the resected metastasis is generally recommended [10].

Importantly, if a radical resection (R0) cannot be technically achieved—whether due to tumor location, patient comorbidities, or extent of disease—the decision to proceed with surgery should be carefully reconsidered. Evidence suggests that non-radical procedures, such as tumor debulking or R2 resections, do not offer a survival benefit and should be avoided in favor of alternative treatments [40,42].

### 4.12. Primary Tumor Location—Colon vs. Rectum

Emerging evidence suggests that the anatomical origin of the primary tumor (colon vs. rectum) may influence prognosis after pulmonary metastasectomy. Rectal cancers tend to be associated with a higher risk of lung recurrence compared with colon cancers, possibly due to differences in venous drainage and metastatic spread patterns. Worse overall survival has been observed in patients with rectal primary tumors undergoing pulmonary metastasectomy compared with those with colon primaries, although results across studies remain heterogeneous [29,30,43].

## 5. Lung Metastasis Surgery

The surgical management of pulmonary metastases from colorectal cancer (CRC) involves a variety of techniques designed to achieve optimal oncological outcomes while minimizing morbidity. The two primary approaches are open thoracotomy and video-assisted thoracoscopic surgery (VATS), each offering distinct advantages and are influenced by specific clinical factors. Open thoracotomy provides extensive exposure of the thoracic cavity and enables direct manual palpation of the lung parenchyma to identify occult metastases not detectable on imaging [9]. This manual examination is particularly valuable in cases with multiple or small nodules, where comprehensive detection is essential for ensuring complete resection. In contrast, VATS—a minimally invasive alternative—offers benefits such as reduced operative trauma, shorter hospital stays, faster recovery, and lower postoperative morbidity [8,9]. Comparative studies have confirmed the oncologic equivalence of VATS and open surgery in well-selected patients, establishing its role as a standard approach for eligible cases [9,44]. However, while the tactile feedback of VATS is limited compared with open thoracotomy, many VATS procedures incorporate a utility incision that allows for some degree of manual palpation. When combined with thin-slice CT imaging, this strategy enables the reliable intraoperative detection of metastases in most cases [11].

Advances in imaging and surgical techniques have expanded the indications for VATS, even for multiple lesions, provided that adequate oncologic resection can be achieved. The choice of surgical approach is influenced by several factors including the number, size, and location of metastases, patient comorbidities, prior thoracic surgeries, and surgeon expertise. Additionally, the emphasis on pulmonary function preservation continues to guide the preference for minimally invasive techniques and parenchyma-sparing resections in appropriate patients [45,46]. Ultimately, metastasectomy should only be pursued when the technical and functional resectability criteria are met, ensuring the possibility of complete (R0) resection without compromising pulmonary function [2,6].

## 6. Systemic Therapies and Surgical Synergy

Although pulmonary metastasectomy remains an important part of managing colorectal cancer (CRC) lung metastases, combining it with systemic treatments, such as chemotherapy and targeted therapies, is essential for improving outcomes. Several studies have demonstrated that perioperative chemotherapy—administered before or after surgical resection—can significantly improve the overall survival (OS) and recurrence-free survival (RFS) in selected patients [7]. This multimodal strategy is particularly valuable for patients with high-risk characteristics, such as multiple metastases or elevated carcinoembryonic antigen (CEA) levels, where systemic therapies enhance disease control beyond what surgery alone can achieve.

A recent meta-analysis from Ratnayake et al. reported comparable survival outcomes between surgical and non-surgical treatments in specific patient populations, emphasizing the need for personalized treatment approaches [47]. Additionally, the role of targeted therapies, guided by molecular markers such as KRAS and BRAF mutations, continues to evolve, with ongoing trials exploring combinations of systemic and local interventions for metastatic CRC [34,48].

In recent years, strategies for the management of pulmonary metastases from colorectal cancer (CRC) have advanced substantially. The adoption of video-assisted thoracoscopic surgery (VATS) has enabled a minimally invasive approach with reduced morbidity and comparable oncological outcomes to open thoracotomy [8,9]. Improved imaging modalities such as PET/CT and enhanced molecular profiling have also refined patient selection for pulmonary metastasectomy (PM), leading to more personalized and effective surgical strategies [1]. In addition to surgery, local ablative modalities such as radiofrequency ablation (RFA) and stereotactic body radiotherapy (SBRT) are increasingly recognized as valuable alternatives in select patients, particularly those unfit for surgery. SBRT offers non-invasive, highly focused treatment with minimal toxicity, especially for small, well-demarcated peripheral lesions. At the same time, local ablative therapies such as radiofrequency ablation (RFA) have emerged as promising alternatives for selected patients, particularly those who are not candidates for surgery. RFA is repeatable, minimally invasive, and associated with a low rate of complications, making it suitable for small, peripheral lesions. Comparative studies suggest that RFA may offer outcomes comparable to PM in carefully selected cases [47].

Beyond local interventions, recent advances in systemic therapy have transformed the treatment landscape for metastatic CRC. Notably, the use of immune checkpoint inhibitors—such as pembrolizumab—has demonstrated impressive and durable responses in patients with microsatellite instability-high (MSI-H) or mismatch repair-deficient (dMMR) tumors. The KEYNOTE-177 trial by Diaz et al. showed that first-line immunotherapy can significantly improve progression-free survival compared with chemotherapy in this subgroup. These findings support the idea that in select molecularly defined populations, systemic therapy alone may offer a meaningful alternative to surgical management [49]. Furthermore, the PulMiCC trial highlighted the need for equipoise in surgical decision-making, challenging previous assumptions regarding the universal survival benefit of PM in all patient groups [50].

Ultimately, the choice among surgery or RFA must be individualized, taking into account factors such as the number and size of metastases, anatomical location, patient comorbidities, prior treatments, and personal preferences. When complete (R0) surgical resection is achievable, and the patient is medically fit, PM remains the treatment of choice, especially when integrated into a multidisciplinary approach that includes systemic therapies [1,8,9,47].

## 7. Recurrent Metastasis

Recurrence of pulmonary metastases following metastasectomy is a frequent clinical challenge in patients with colorectal cancer (CRC), with reported rates ranging from 30% to 68% depending on tumor biology, extent of disease, and initial surgical margins [12,51].While only 10–15% of CRC patients develop lung metastases, a substantial proportion of those undergoing pulmonary metastasectomy (PM) experience re-recurrence. This discrepancy raises important questions regarding the curative potential of PM and whether early recurrence represents true relapse or the progression of micrometastatic disease not detected at the time of initial intervention [2,3,51]. Some authors have proposed that delaying initial PM may help reveal tumor biology, as longer intervals before recurrence could reflect more indolent disease. In carefully selected patients, repeat pulmonary metastasectomy is technically feasible and has been associated with acceptable long-term outcomes. However, the survival benefit of repeat surgery remains uncertain due to the lack of randomized comparisons with non-surgical treatments and the potential influence of lead-time bias. For instance, the study by Ihn et al. reported a 5-year survival rate of approximately 58% for patients undergoing repeat metastasectomy, but it did not include a comparator group of patients managed non-operatively, limiting the strength of the conclusions [51].

However, reoperation decisions should be guided by strict selection criteria including factors such as:

- Disease-free interval (DFI) after the initial metastasectomy;
- Number and location of recurrent lesions;
- Pulmonary reserve and overall functional status;
- Absence of extrapulmonary metastases [43,51].

A multidisciplinary tumor board approach is essential to weigh the risks and benefits of repeat surgery, taking into account alternative treatments such as stereotactic body radiotherapy (SBRT), systemic chemotherapy, or targeted therapies for patients who are unfit for surgery [30].Notably, in selected patients with microsatellite instability-high (MSI-H) or mismatch repair-deficient (dMMR) tumors, checkpoint inhibitors such as pembrolizumab have demonstrated an over 50% 5-year overall survival without surgery, as shown in the KEYNOTE-177 trial by Diaz et al. [49].

Furthermore, close postoperative surveillance with regular imaging and CEA monitoring remains critical for the early detection of recurrences, ensuring that timely interventions can be made to optimize patient outcomes [12].

## 8. Survival Outcomes Across Studies

The literature reports favorable long-term outcome survival following pulmonary metastasectomy for colorectal cancer, though with significant variability across studies. Differences in patient selection criteria, tumor biology, and surgical techniques contribute to this heterogeneity. The reported five-year survival rates range from approximately 40% to over 70%, reflecting both advances in surgical approaches and the influence of prognostic factors [3,4,22].

Table 1 presents a selection of key studies evaluating the five-year survival outcomes, revealing the diversity of findings. Factors such as resection margins, primary tumor site, lymph node involvement, and molecular markers like KRAS mutations have been identified as important predictors of survival in this context [10,12,48]. This variation emphasizes the critical need for individualized treatment strategies and multidisciplinary evaluation in managing patients undergoing lung metastasectomy.

## 9. Long-Term Outcomes and Challenges

Lung metastasectomy for colorectal cancer (CRC) has demonstrated promising long-term outcomes in appropriately selected patients, with perioperative mortality rates reported to be low, typically ranging between 0% and 0.5% [4,12]. However, despite these encouraging survival metrics, challenges persist, particularly regarding intrathoracic recurrence. Recurrence rates after pulmonary metastasectomy can reach as high as 30% to 68%, depending on factors such as tumor biology, resection margins, lymph node involvement, and disease-free interval (DFI) [12,51]. Local recurrence within the lungs remains the most common pattern, often necessitating additional therapeutic interventions such as repeat metastasectomy, stereotactic body radiotherapy (SBRT), or systemic treatments [31,43].

Despite these recurrence challenges, metastasectomy continues to offer durable disease control and enhanced survival outcomes for a subset of patients with oligometastatic CRC. The reported 5-year overall survival (OS) rates after pulmonary resection range widely from 27% to 68%, influenced by patient selection, extent of disease, and surgical radicality [4,12]. Emerging data emphasize the need for long-term follow-up and multimodal management strategies to address recurrent disease and to maximize patient benefit. Additionally, current research into biomarkers (e.g., KRAS/BRAF status, CEA dynamics) and molecular imaging techniques may further refine patient selection and personalized treatment approaches in the future [28,48].

## 10. Limitations

Despite the promising outcomes associated with lung metastasectomy in colorectal cancer patients, several limitations must be considered. The heterogeneity of the study designs, patient populations, and treatment protocols across the literature presents challenges in directly comparing outcomes and establishing universal treatment guidelines. The possibility for selection bias is also present, as many studies are retrospective in nature, and patient eligibility for surgery is influenced by clinical judgment and institutional practices.

Furthermore, the PulMiCC trial series has raised significant debate regarding the generalized benefit of pulmonary metastasectomy, as randomized controlled data did not demonstrate a clear survival or quality of life advantage over non-surgical management in selected cohorts [50,62,63,64,65]. However, the applicability of these findings remains a subject of active debate, especially considering the patient selection criteria and trial limitations. These findings point to the importance of careful patient selection and reinforce the necessity for prospective, randomized studies. Such trials could stratify patients by molecular characteristics (e.g., MSI or RAS/BRAF status) and incorporate contemporary therapies like checkpoint inhibitors, aiming to clarify which subgroups benefit most from surgery. Finally, although surgical resection continues to play a central role in treatment, the long-term efficacy of metastasectomy combined with novel systemic therapies has yet to be fully explored in well-controlled studies.

## 11. Conclusions

Lung metastasectomy represents one of several potential components of the multimodal treatment strategy for colorectal cancer patients with resectable pulmonary metastases, offering a possible curative approach for selected patients with limited, isolated metastatic disease. While advancements in systemic therapies, including chemotherapy, targeted treatments, and immunotherapy, have improved overall management, the role of surgical resection should be carefully individualized and considered in the context of multidisciplinary decision-making. This is particularly evident in patients with favorable prognostic factors, such as solitary metastasis and a long disease-free interval, where metastasectomy may significantly impact the long-term survival.

This review highlights several additional key prognostic variables, including molecular markers (e.g., KRAS, BRAF), lymph node involvement, response to systemic therapy, and performance status, that influence both the patient selection and expected outcomes. For example, patients with KRAS or BRAF mutations tend to have worse prognoses, while those demonstrating a good response to chemotherapy may derive a greater benefit from surgery. Moreover, anatomical factors such as bilateral disease or multiple lesions require individualized assessment, particularly in light of resectability and pulmonary reserve.

By integrating these diverse clinical, pathological, and molecular factors into surgical planning, clinicians can better identify candidates most likely to benefit from pulmonary metastasectomy and avoid unnecessary interventions in those with poor prognostic profiles.

## Figures and Tables

**Table 1:** Summary of key studies reporting the five-year survival outcomes following pulmonary metastasectomy in colorectal cancer patients.

Study	Year	Number of Patients	5-Year Survival	Patient Characteristics	Contribution	Limitations	Key Clinical Insights
Gao et al. [52]	2024	120	72%	Selected patients with single or few metastases; CEA monitoring; most received perioperative chemotherapy.	Highlighted both survival benefit and recurrence risk post-PM, emphasizing need for patient selection and long-term monitoring.	Retrospective, single-center; no control group; potential selection bias.	Longer disease-free interval and solitary lesions predict better survival.
Denz et al. [30]	2024	418	81.2%	Median age 65; mainly 1–3 nodules; R0 resections; no MSI/MMR data reported.	Demonstrated a significant survival advantage with PM over non-surgical care, reinforcing surgical value in selected cases.	Retrospective study; no comparison with non-surgical treatment.	Repeat metastasectomy feasible with 58% 5-yr OS; selection is critical.
Carvajal et al. [13]	2022	82	33.2%	Younger patients; some with extrapulmonary disease; low disease volume overall.	Provided real-world survival outcomes from a South American cancer center, offering insight into feasibility and effectiveness of PM in LMIC settings.	Retrospective, single-institution; limited generalizability.	Surgical intent leads to improved OS; patient comorbidities impact outcomes.
Gössling et al. [18]	2021	58	49.80%	ECOG 0–1; 1–3 pulmonary lesions; most had preoperative chemotherapy.	Reported a 5-year survival of 49.8%, supporting curative-intent pulmonary metastasectomy as a viable option in selected mCRC patients.	Retrospective, small sample; selection bias possible.	Curative resection linked to improved survival in real-world practice.
Sponholz et al. [53]	2021	233	47%	Left vs. right primary tumor location; limited number of metastases; molecular status not reported.	Demonstrated that primary tumor location significantly impacts survival outcomes after lung metastasectomy for CRC.	Retrospective data; no randomization or comparison group.	Primary tumor location influences post-metastasectomy survival.
Vidarsdottir et al. [54]	2021	216	56%	~40% KRAS mutant; elevated preoperative CEA; all had complete lung resections.	Analyzed surgically treated CRC lung metastases and highlighted the prognostic relevance of tumor biology in survival outcomes.	Retrospective; small cohort; lacked molecular stratification.	CEA and KRAS mutation status affect prognosis post-metastasectomy.
Davini et al. [10]	2020	210	54%	Majority with solitary lesions; older age group; good performance status.	Showed that negative resection margins are strongly associated with improved long-term survival post-metastasectomy.	Retrospective analysis; surgical candidates only; no systemic therapy control.	Pulmonary resection feasible even in elderly with good selection.
Vodička et al. [55]	2020	104	54.30%	Solitary lesions; R0 resections; patients without major organ dysfunction.	Analyzed prognostic factors and outcomes, supporting metastasectomy as a valid treatment in multimodal CRC management.	Retrospective design; potential lead-time and selection biases.	Good outcomes achieved after resection of solitary metastases.
Huang et al. [29]	2020	179	40.80%	Variable primary tumor locations; clinically stable; preoperative therapy not detailed.	Emphasized the prognostic relevance of primary tumor location in CRC patients undergoing lung metastasectomy.	Small sample size; retrospective; no standard criteria for surgery.	Right-sided primary tumors associated with better outcomes.
Corsini et al. [56]	2020	194	57%	DFI > 12 months common; ~50% VATS; few underwent repeat resections.	Revealed significant survival differences depending on whether the primary tumor was right- or left-sided.	Retrospective; lacked uniform treatment protocols.	VATS resection is safe and effective; short DFI predicts recurrence.
Rapicetta et al. [57]	2019	344	61.90%	Solitary metastasis predominant; good functional status; frequent adjuvant therapy use.	Assessed the value of adjuvant chemotherapy after resection of a single metastasis, indicating possible reduction in recurrence.	Retrospective; no comparison to non-surgical alternatives.	Repeat metastasectomy offers durable outcomes in oligometastatic patients.
Renaud et al. [58]	2019	574	58%	Good general health; moderate metastatic burden; low NLR associated with benefit.	Found that elevated neutrophil-to-lymphocyte ratio is linked to worse prognosis in lung metastasectomy for CRC.	Retrospective; surgical approach varied between centers.	Multicenter data support surgery in selected metastatic CRC cases.
Nanji et al. [3]	2018	420	40%	45% with multiple lesions; comorbidities recorded; adequate pulmonary reserve.	Highlighted key predictors of survival in real-world metastasectomy practice, including margin status and comorbidity burden.	Single-center; lacked molecular profiling data.	Worse prognosis in patients with multiple heterogeneous lesions.
Al-Ameri et al. [4]	2018	756	56%	Middle-aged; typically 2 lesions; R0 resections in most cases.	Identified several clinicopathological factors influencing survival, supporting surgical intervention in selected patients.	Retrospective; outcomes possibly influenced by surgical technique.	Multiple surgeries do not compromise long-term survival.
Fournel et al. [59]	2017	306	59%	Solitary or double peripheral lesions; all treated with curative intent.	Confirmed the prognostic value of complete resection and highlighted recurrence risks even after radical surgery.	Selection bias; heterogeneous patient population.	Molecular profiling may guide future patient selection.
Sun et al. [60]	2017	154	71.30%	All underwent VATS; lesions < 3 cm; limited metastatic spread.	Demonstrated favorable outcomes of VATS approach in CRC lung metastasectomy with a high 5-year survival rate.	Retrospective cohort; unclear systemic treatment details.	Surgical margin status key for long-term survival.
Karim et al. [61]	2017	377	40%	All had resected primary CRC; good performance status; molecular profile unclear.	Evaluated the use of chemotherapy post-metastasectomy, showing real-world trends and variable outcome benefits.	No survival benefit in adjusted analysis; matched control group.	RCT (PulMiCC) challenges survival benefit of PM in unselected patients.
Yokoyama et al. [38]	2017	59	54.30%	KRAS/BRAF status known; modern chemotherapy use; mostly solitary lung lesions.	Evaluated outcomes of initial lung metastasectomy in mCRC patients, demonstrating favorable survival in the era of modern chemotherapy.	Retrospective; KRAS/BRAF status not uniformly reported.	KRAS/BRAF status linked to survival post-metastasectomy.
